# Mesenchymal-Epithelial Transition Exon 14 Skipping Mutation and Amplification in 5,008 Patients With Lung Cancer

**DOI:** 10.3389/fonc.2021.755031

**Published:** 2021-09-30

**Authors:** Yaolin Song, Guangqi Li, Kun Ju, Wenwen Ran, Han Zhao, Xianglan Liu, Mingyu Hou, Yulu He, Yang Chen, Guoliang Zang, Xiaoming Xing

**Affiliations:** ^1^ Department of Pathology, The Affiliated Hospital of Qingdao University, Qingdao, China; ^2^ Department of Emergency, Affiliated Hospital of Qingdao University, Qingdao, China; ^3^ Department of Technical, Geneis Beijing Co., Ltd., Beijing, China

**Keywords:** lung cancer, MET exon 14 skipping mutation, MET amplification, MET DNA splicing sites, clinical character

## Abstract

**Background:**

Lung cancer is a major health concern worldwide because of its increasing incidence and mortality. This study aimed to clarify the association between mesenchymal-epithelial transition (MET) genomic alterations and clinical characteristics of lung cancer.

**Method:**

We collected data from 5,008 patients with lung cancer diagnosed and treated between January 2017 and July 2021 at the Affiliated Hospital of Qingdao University. Genomic alterations in the MET gene, including the exon 14 skipping mutation and amplification, were detected using amplification refractory mutation system-polymerase chain reaction (2,057 cases) and next-generation sequencing (2,951 cases). Clinical characteristics such as age, sex, tumor location, tumor stage, smoking, pleural invasion, and histology were statistically analyzed for MET exon 14 skipping mutation and amplification. The DNA splicing sites causing the MET exon 14 skipping mutation at the mRNA level were also investigated.

**Results:**

The incidence of the MET exon 14 skipping mutation was 0.90% (41/4,564) in adenocarcinoma, 1.02% (3/294) in squamous cell carcinoma, and 8.33% (1/12) in sarcomatoid carcinoma specimens. It was more frequently observed in patients over 60 years of age than the MET exon 14 skipping mutation wildtype. The MET exon 14 skipping mutation co-occurred with epidermal growth factor receptor (EGFR) L858R, EGFR 19-Del, and BRAF V600E mutations. At the DNA level, single nucleotide mutation and small fragment deletion (1–38 base pairs) upstream and downstream of MET exon 14 led to MET exon 14 skipping mutation at the mRNA level. MET amplification occurred in 0.78% (21/2,676) adenocarcinoma and 1.07% (2/187) squamous cell carcinoma specimens and was significantly associated with advanced tumor stages (III + IV) compared to the MET amplification wildtype. MET amplification primarily co-occurred with the EGFR mutation.

**Conclusions:**

Our study found that MET genomic alterations were statistically related to age and tumor stage and co-existed with mutations of other oncogenic driver genes, such as EGFR and BRAF. Moreover, various splicing site changes at the DNA level led to the exon 14 skipping mutation at the mRNA level. Further studies are required to clarify the association between MET genomic alterations and prognosis.

## Introduction

Lung cancer is the leading cause of tumor-related deaths worldwide. In China, the incidence and mortality of lung cancer have increased rapidly in recent years (1, 2). Several risk factors, including smoking, genetic alterations, air pollution, and chronic obstructive pulmonary disease, contribute to the development of lung cancer (1). Oncogenic driver gene mutations, such as those in epidermal growth factor receptor (EGFR), KRAS, ALK, human epidermal growth factor receptor 2 (HER2), and mesenchymal-epithelial transition (MET), play essential roles in the initiation, progression, and clinical treatment of lung cancer.

MET is a tyrosine kinase receptor for the hepatocyte growth factor ligand. Aberrant activation of the MET signaling pathway is closely associated with the development of several solid tumors and can be caused by MET exon 14 skipping mutation, MET amplification, and overexpression of hepatocyte growth factor or MET (3, 4). Somatic mutations in the MET gene can result in MET exon 14 skipping mutation, generating an incomplete MET receptor that lacks the tyrosine 1003 binding site for Cbl, and leading to sustained MET signaling pathway activation and increased oncogenic potential (5). MET exon 14 skipping mutation occurs in approximately 3% of lung adenocarcinoma and 2.3% of other lung cancer subtypes and is mutually exclusive with other known driver gene mutations such as EGFR, KRAS, and HER2, suggesting its potential as a true oncogenic driver site (6, 7). In non-small cell lung cancer (NSCLC), MET exon 14 skipping mutation is more closely related to females, former or current smokers, and poor prognosis than the MET wildtype (8). MET exon 14 is more frequently found in older patients than in patients with EGFR- or KRAS-mutant lung cancer (9). DNA-based sequencing, such as next-generation sequencing (NGS), and RNA-based sequencing, such as amplification refractory mutation system-polymerase chain reaction (ARMS-PCR), can both detect MET exon 14 skipping alterations (10, 11).

MET amplification occurs in approximately 1–6% of patients with NSCLC (12). High-level MET amplification is mutually exclusive of other driver mutations, except MET mutation, whereas low-level MET amplification can co-occur with EGFR or KRAS mutations (7). MET amplification is an important acquired resistance mechanism to first- or second-generation EGFR-tyrosine kinase inhibitors (TKIs) based on T790M mutation (13), accounting for approximately 10–20% of the acquired resistance to TKIs in EGFR-mutant NSCLC (14). MET amplification is more frequent in patients who smoke (7) and in advanced tumor stage and solid predominant subtype of adenocarcinoma in patients with EGFR wildtype lung cancer (15). It can be detected by fluorescence *in situ* hybridization and NGS (16). Furthermore, both MET exon 14 skipping mutation and MET amplification are independent factors of poor prognosis in lung cancer (17, 18).

Although MET has shown strong oncogenic potential and is prevalent in lung cancer, therapeutics targeting MET overexpression have been unsatisfactory to date. Recently, MET exon 14 skipping and high-level MET gene amplification have been considered as predictive biomarkers for MET inhibition in patients with NSCLC (19–22). Combining MET inhibitors with other target drugs, such as EGFR-TKIs, might represent promising therapeutic effects in specific subgroups of patients (23). Based on preliminary data and FDA approval, the National Comprehensive Cancer Network has recommended several TKIs such as crizotinib, capmatinib, and tepotinib as first-line therapy or subsequent therapy options for patients with NSCLC (24).

In our study, we collected data on 5,008 cases of diverse lung tumor types at our medical center, detected the MET gene alterations by NGS and ARMS-PCR, and analyzed the clinical, molecular, and pathological characteristics of these patients. We used NGS and ARMS-PCR to verify the DNA splicing sites that could cause the MET exon 14 skipping mutation at the mRNA level.

## Materials and Methods

### Patients and Study Design

We collected data from 5,564 cases of various lung cancer types that were diagnosed and medically treated at the Affiliated Hospital of Qingdao University between January 2017 and July 2021. Patients who refused to participate in the present study (217 cases) and used blood samples for gene testing (339 cases) were excluded from the study. In total, 5,008 lung cancer cases were analyzed. For patients who developed more than one lung tumor, the more advanced tumor was selected for this study. All patients provided written informed consent, and the Ethics Committee of Qingdao University approved the study (approval NO. QYFY WZLL 26577).

### Next-Generation Sequencing

Tissues were fixed with 4% paraformaldehyde, and genomic DNA was extracted using a Tiangen paraffin-embedded tissue DNA extraction kit (Tiangen Biotech, Beijing, China) following the manufacturer’s instructions. The DNA quality was assessed using a Qubit dsDNA assay kit (Invitrogen, Carlsbad, CA, USA) according to the manufacturer’s instructions. Libraries were prepared using the human 10 gene mutation detection kit (Genesis, Beijing, China) and captured using a gene probe pool. NGS was conducted on an Illumina MiniSeq System (Illumina, San Diego, USA) using a MiniSeq High Output Reagent Kit (Illumina) following the manufacturer’s instructions. Gene mutations were analyzed using Crest, ionCOPY, FreeBay, and Annoval.

For MET mutation, the probe covers all exons and intron 13 of the MET gene and could detect the single nucleotide variations (SNVs) and indel mutations in this interval. MET mutation was determined when the sequencing depth was ≥500X and the mutation frequency was ≥1%. For MET amplification, the probe was separated into 22 segments, which cover all 21 exons and a part of intron 2. Sequencing data was calculated using the copy number (CN) value for each segment; CN ≥ 3.48 was defined as positive for each segment. When more than 90% of the segments are positive, it is interpreted as MET amplification.

The quality of the NGS method was controlled in the following four ways: (1) FFPE samples prepared within 2 years and the proportion of tumor cells over 20% were used. (2) The total amount of DNA extracted should be over 200 ng, and the main band of the extracted DNA detected by gel electrophoresis should be above 500 bp. (3) After the hybridization library is purified, library concentration should be between 1 and 10 ng/μl and the average fragment size should be within 300–500 bp; and (4) the average sequencing depth of each sample should be ≥500X and the coverage over 95%.

### Amplification Refractory Mutation System-Polymerase Chain Reaction

RNA was extracted from paraffin-embedded lung cancer tissues using an FFPE DNA/RNA extraction kit (AmoyDx, Xiamen, China). The MET exon 14 skipping mutation was detected using the human lung cancer multi-gene mutation detection kit (fluorescence PCR method) (AmoyDx, Xiamen, China), which includes a probe covering a sequence connecting exons 13 and 15 splicing genes and exons. ARMS-PCR was conducted on an ABI 7500 platform (Applied Biosystems, MA, USA), and the running protocol was set up as follows: 5 min at 42°C and 5 min at 95°C; 25 s at 95°C, 20 s at 72°C for 10 cycles; 25 s at 93°C, 20 s at 72°C for 36 cycles; and maintained at 4°C. The FAM signal represents whether the MET gene in the sample RNA is amplified, and the HEX signal represents the MET gene expression. MET mutation was determined when the FAM signal Ct value ≤27 and △Ct value (FAM Ct - HEX Ct) ≤6. The quality control of ARMS-PCR method includes two parts: (1) FFPE samples within 2 years and the presence of tumor cells; and (2) the OD_260_/OD_280_ of the extracted RNA should be within 1.8–2.1, and the concentration of RNA should be 20–500 ng/μl.

### Statistical Analysis

All data were analyzed using SPSS 26.0.0 statistical analysis software (IBM Corp., Armonk, NY, USA). The association between different groups (MET amplification *versus* MET amplification wildtype; MET exon 14 skipping *versus* MET exon 14 skipping mutation wildtype) was evaluated using the standard Chi-square test. Data that were not qualified for the Chi-square test were merged into the groups to reach the standard. Statistical significance was set at *P* < 0.05.

## Results

### Patient Characteristics

The basic clinical characteristics of the 5,008 patients are summarized in **Table 1**. A total of 2,057 and 2,951 patients were tested using ARMS-PCR and NGS, respectively. Tissue types included 3,774 surgical resection specimens, 1,161 needle biopsy specimens, and 73 pleural or pericardial effusion specimens. Among the 5,008 patients, 2,248 (44.89%) were males and 2,760 (55.11%) were females, with an average age of 60.5 years (range = 16–94 years). Among the 5,008 lung cancer cases, 7 had an unknown smoking history, 1,572 (31.43%) were former/current smokers, and 3,429 (68.57%) were non-smokers. Tumor staging was performed according to the 8th edition of the American Joint Committee on Cancer Staging Manual (25); 14 cases (0.29%), 3,202 cases (65.55%), 624 cases (12.77%), 259 cases (5.30%), and 786 cases (16.09%) were in stages 0, I, II, III, and IV, respectively. The pathological types of these patients were mostly adenocarcinoma (4,564 cases, 92.11%), squamous cell carcinoma (294 cases, 5.93%), adenosquamous carcinoma (33 cases, 0.67%), neuroendocrine carcinoma (32 cases, 0.65%), salivary gland-type tumors (12 cases, 0.24%), sarcomatoid carcinoma (12 cases, 0.24%), large cell carcinoma (6 cases, 0.12%), sarcoma (1 case, 0.02%), and undifferentiated carcinoma (1 case, 0.02%).

**Table 1:** Basic clinical information of 5,008 cases of patients with lung cancer.

Clinical characteristics	Number of patients (%)
**Age**	**N = 5,008**
>60 years	2,569 (51.30)
≤60 years	2,439 (48.70)
**Sex**	**N = 5,008**
Male	2,248 (44.89)
Female	2,760 (55.11)
**Tumor location**	**N = 4,652**
Left	1,936 (41.62)
Right	2,716 (58.38)
**Smoke**	**N = 5,001**
Ever	1,572 (31.43)
Never	3,429 (68.57)
**AJCC tumor stage**	**N = 4885**
0	14 (0.29)
I	3,202 (65.55)
II	624 (12.77)
III	259 (5.30)
IV	786 (16.09)
**Histology type**	**N = 4,955**
Adenocarcinoma	4,564 (92.11)
Squamous cell carcinoma	294 (5.93)
Adenosquamous carcinoma	33 (0.67)
Neuroendocrine carcinoma	32 (0.65)
Salivary gland-type tumors	12 (0.24)
Sarcomatoid carcinoma	12 (0.24)
Large cell carcinoma	6 (0.12)
Sarcoma	1 (0.02)
Undifferentiated carcinoma	1 (0.02)

bold values indicate the number of total patients in each clinical characters that were analyzed.

### Characterization of the MET Exon 14 Skipping Mutation

NGS and ARMS-PCR detected the MET exon 14 skipping mutation. We found 45 patients with the MET exon 14 skipping mutation among the 5,008 cases, and the mutation rate was 0.91% (45/4,955). Of these 45 cases, 27 were detected using ARMS-PCR, and 18 were tested with NGS. For different histological types, the incidence was 0.90% (41/4,564) in adenocarcinoma, 1.02% (3/294) in squamous cell carcinoma, and 8.33% (1/12) in sarcomatoid carcinomas. The association between the MET exon 14 skipping mutation and clinical characteristics is summarized in **Table 2**. We found that MET exon 14 skipping mutation was significantly associated with older age (*P* = 0.001), and there was no measurable significance between MET exon 14 skipping and MET exon 14 skipping wildtype for sex, tumor stage, tumor location, smoking, histology type, and pleural invasion.

**Table 2:** Clinicopathological characteristics and MET exon 14 skipping mutation.

Pathological characteristics	Total number (%)	MET ex14 skipping MT (%)	MET ex14 skipping WT (%)	*P* value (Mut *vs* WT)
**Age**	**5,008**	**45**	**4,963**	**0.001**^**a**^^,^*
>60 years old	2,569 (51.30)	34 (1.32)	2,535 (98.68)	
≤60 years old	2,439 (48.70)	11 (0.45)	2,428 (99.55)	
**Sex**	**5,008**	**45**	**4,963**	**0.588**^**a**^
Male	2,248 (44.89)	22 (0.98)	2,226 (99.02)	
Female	2,760 (55.11)	23 (0.83)	2,737 (99.17)	
**AJCC tumor stage**	**4,885**	**45**	**4,840**	**0.553**^**a**^
0+I+II	3,840 (78.61)	37 (0.96)	3,803 (99.04)	
III+IV	1,045 (21.39)	8 (0.77)	1,037 (99.23)	
**Tumor location**	**4,652**	**45**	**4,607**	**0.257**^**a**^
Left	1,936 (41.62)	15 (0.77)	1,921 (99.23)	
Right	2,716 (58.38)	30 (1.10)	2,686 (98.90)	
**Pleural invasion**	**3,598**	**35**	**3,563**	**0.854**^**a**^
Yes	660 (18.34)	6 (0.91)	654 (99.09)	
No	2,938 (81.66)	29 (0.99)	2,909 (99.01)	
**Smoke**	**5,001**	**45**	**4,956**	**0.712**^**a**^
Ever	1,572 (31.43)	13 (0.83)	1,559 (99.17)	
Never	3,429 (68.57)	32 (0.93)	3,397 (99.07)	
**Histology type**	**4,955**	**45**	**4,910**	**1.000**^**a,#**^
Adenocarcinoma	4,564 (92.11)	41 (0.90)	4,523 (99.10)	
Squamous cell carcinoma	294 (5.93)	3 (1.02)	291 (98.98)	
Other types	97 (1.96)	1 (1.03)	96 (98.97)	

a Pearson’s chi-squared test; ^#^Significance between adenocarcinoma and squamous cell carcinoma.

*Statistically significant.

MT, Mutant; WT, Wildtype; ex14, exon 14.

bold values indicate the number of total patients in each clinical characters that were analyzed.

### MET Amplification Analysis

We used NGS to detect MET amplification and found that among the 2,951 cases, 23 patients developed MET amplification, with an occurrence rate of 0.79% (23/2,927). For different histological types, the mutation rate was 0.78% (21/2,676) adenocarcinoma and 1.07% (2/187) squamous cell carcinoma. The relationship between MET amplification and clinical characteristics is shown in **Table 3**. MET amplification was significantly associated with advanced tumor stage (phase 0 + I + II *versus* phase III + IV, *P* = 0.000). There was no measurable difference between MET amplification and MET amplification wildtype for age, sex, smoking history, histology type, and pleural invasion. Moreover, MET amplification exhibited a trend of higher incidence in the right lobe of the lung (left *versus* right, *P* = 0.058).

**Table 3:** Clinicopathological characteristics and MET amplification.

Pathological characteristics	Total number (%)	MET amplification (%)	MET amplification WT (%)	*P* value (Amp *vs* Amp WT)
**Age**	**2,951**	**23**	**2,928**	**0.318**^**a**^
>60 years old	1,490 (50.49)	14 (9.40)	1,476 (90.60)	
≤60 years old	1,461 (49.51)	9 (0.62)	1,452 (99.38)	
**Sex**	**2,951**	**23**	**2,928**	**0.104**^**a**^
Male	1,302 (44.12)	14 (1.08)	1,288 (98.92)	
Female	1,649 (55.88)	9 (0.55)	1,640 (99.45)	
**AJCC tumor stage**	**2,860**	**23**	**2,837**	**0.000**^**a**^^,^*
0+I+II	2,321 (81.15)	10 (0.43)	2,311 (99.57)	
III+IV	539 (18.85)	13 (2.41)	526 (97.59)	
**Tumor location**	**2,880**	**23**	**2,857**	**0.058**^**a**^
Left	1,185 (41.15)	5 (0.42)	1,180 (99.58)	
Right	1,695 (58.85)	18 (1.06)	1,677 (98.94)	
**Pleural invasion**	**2,356**	**10**	**2,346**	**0.780**^**a**^
Yes	434 (18.42)	1 (0.23)	433 (99.77)	
No	1,922 (81.58)	9 (0.47)	1,913 (99.53)	
**Smoke**	**2,945**	**21**	**2,924**	**0.492**^**a**^
Ever	918 (31.17)	8 (0.87)	910 (99.13)	
Never	2,027 (68.83)	13 (0.64)	2,014 (99.36)	
**Histology type**	**2,927**	**23**	**2,904**	**1.000**^**a,#**^
Adenocarcinoma	2,676 (91.42)	21 (0.78)	2,655 (99.22)	
Squamous cell carcinoma	187 (6.39)	2 (1.07)	185 (98.93)	
Other types	64 (2.19)	0 (0.00)	64 (100.00)	

a Pearson’s chi-squared test; ^#^Significance between adenocarcinoma and squamous cell carcinoma.

*Statistically significant.

MT, Mutant; WT, Wildtype; Amp, Amplification.

bold values indicate the number of total patients in each clinical characters that were analyzed.

### Co-Mutation of MET With Other Oncogenic Driver Genes

MET amplification is an important resistance mechanism of TKIs in EGFR-mutant NSCLC. However, in our study, we found 11 patients with primary MET amplification and EGFR mutation. Unfortunately, all patients underwent surgical resection and remained stable, making it impossible to track the therapeutic effect of TKIs on these patients in the short term. Usually, MET exon 14 skipping is mutually exclusive with other oncogenic driver genes, such as EGFR (19). However, in our study, we found three cases of patients harboring concurrent mutations with MET exon 14 skipping, including EGFR exon 21 L858R, EGFR exon19 19-Del, and BRAF exon 15 V600E. We also reported one case that harbored both MET amplification and MET exon 14 c.3027_3028+9del mutation. All histological types of these patients were adenocarcinoma. The clinical information and co-existing gene alterations in these patients are summarized in **Table 4**.

**Table 4:** Co-mutations and clinical information of patients with MET genomic alterations.

Case No.	Sex	Age (year)	MET alteration	Co-mutations	Tumor stage	Tumor location	Smoke	Pleural invasion
1	M	56	Amplification	EGFR exon20 c.2361G>A	IIIB	L	Y	N/A
2	M	60		EGFR exon20 p.Val769_Asp770insAlaSerVal	IA1	R	N	N
3	M	67		EGFR exon21 p.L858R	IA3	R	N	N
4^#^	M	49		EGFR exon19 p.Glu746_Ala750del; PIK3CA exon20 p.Asn1044Ser	IV	R	N	N
5	F	58		EGFR exon21 p.L858R	IV	R	N	N/A
6	F	63		EGFR exon19 p.Glu746_Ala750del	IA1	R	N	N
7	M	75		EGFR exon19 p.Glu746_Ala750del	IV	R	N	N/A
8	F	48		EGFR exon19 p.Glu746_Ala750del	IV	R	N	N/A
9	M	64		EGFR exon19 p.Glu746_Ala750del	IV	L	Y	N/A
10^*^	F	38		EGFR exon19 p.Glu746_Ala750del;	IIIA	R	N	N
				KRAS exon2 p.G12C;				
				HER2 Amplification				
11	F	58		EGFR exon19 p.Leu747_Thr751del;	IV	R	N	N/A
				EGFR exon20 p.T790M;				
				EGFR exon20 p.C797S				
12	F	70		EGFR exon21 p.L858R	IA1	R	N	N/A
13	M	57		EGFR exon21 p.L858R	IIB	R	N	Y
14	M	73		MET exon14 c.3027_3028+9del	IIA	R	Y	N
15	F	79	Exon 14 skipping	EGFR exon19 19-Del	IA1	L	N/A	N
16	M	79		BRAF exon15 V600E	IV	L	N/A	N
17	M	62		EGFR exon21 L858R	IA1	R	N	N

M, male; F, female; N/A, not available; N, no; Y, yes; R, right; L, left.

^#^Acquired mutation after using gefitinib; ^*^Acquired mutation after using icotinib.

### MET DNA Splicing Sites That Lead to Exon 14 Skipping Mutation

We cross-checked the MET exon 14 skipping patients using ARMS-PCR and NGS to analyze the DNA splicing sites that could cause the MET exon 14 skipping mutation at the mRNA level. Among the 45 cases of MET exon 14 skipping mutation, 7 patients refused to test again, DNA extracted from 3 samples were not qualified for NGS detection requirement, and 35 cases were cross-checked. The DNA splicing sites that could cause mRNA level MET exon 14 skipping and the clinical information of each case are summarized in **Table 5**. Briefly, SNV and small fragment deletion (1–38 base pairs [bp]) led to MET exon 14 skipping. SNV mostly occurred in the region of the upstream and downstream splicing site of exon 14 ± 2 bp, and deletion occurred both upstream and downstream of exon 14, and the deleted fragment usually contained the splicing sites.

**Table 5:** DNA splicing sites and clinical information of patients with the MET exon 14 skipping mutation.

Case no.	Sex	Age (year)	DNA splicing sites	Histology type	Tumor stage	Tumor location	Smoke	Pleural invasion
1	M	61	c.2888-1 G>A	AD	IA2	R	N	Y
2	M	66	c.2888-13_2908del	AD	IV	R	N/A	Y
3	F	64	c.2888-42_2892del	AD	0	R	N	N
4	M	75	c.2942-44_2942-11delinsAAGTCTC	AD	IB	L	Y	Y
5	M	81	c.3004_3028+5del	SCC	IA3	L	N/A	Y
6	F	56	c.3025_3028+2del	AD	IB	R	N	N
7	M	73	c.3027_3028+9del	AD	IIA	R	N	Y
8	F	59	c.3028+2_3028+16del	AD	IIB	R	N	N
9	F	67	c.3028+2delinsAA	AD	IB	L	Y	N
10	M	79	2888-17_2888-2del	AD	IV	L	N	N/A
11	F	73	c.2888-20_2888-4del	AD	IB	R	N	N
12	F	60	c.2888-20_2888-9del	AD	IA1	R	N	N
13	F	59	c.2888-30_2888-15del	AD	IA1	L	N	N
14	F	48	c.2888-38_2888-1del	AD	IA1	R	N	N
15	F	61	c.2888-6_2907del	AD	IA3	R	N	N
16	F	79	c.3014-3027del	AD	IA3	R	N	N
17	F	54	c.3022-3028+7del	SCC	IIIB	L	N	N/A
18	M	62	c.3025_3028+5del	SCC	IV	L	N	N/A
19	M	61	c.3028+1G>A	AD	IA1	R	N	N
20	F	76	c.3028+1G>A	AD	IA1	R	N	Y
21	M	71	c.3028+1G>A	AD	IA3	L	N	Y
22	M	63	c.3028+1G>T	AD	IB	R	N	N
23	M	65	c.3028+1G>T	AD	IA2	R	N	Y
24	F	73	c.3028+2T>A	AD	IA1	R	N	N
25	M	57	c.3028+3A>T	AD	IIA	L	N	Y
26	M	70	c.3028+3A>T	AD	IA3	R	N	N
27	F	72	c.3028G>A	AD	IIB	R	N	Y
28	M	54	c.3028G>A	AD	IA2	L	N	Y
29	F	52	c.3028G>C	AD	IA1	R	N	N
30	F	71	c.3028G>C	AD	IA2	L	N	N
31	F	67	c.3028G>C	AD	IA2	R	N	N
32	M	66	c.3028G>C	AD	IA3	R	N	N
33	M	63	c.3028G>T	AD	IA1	L	Y	N
34	F	74	c.3028G>T	AD	IIA	R	N	N
35	M	64	c.3028G>T	SC	IB	L	N	Y

M, male; F, female; AD, adenocarcinoma; SCC, squamous cell carcinoma; SC, sarcomatoid carcinoma; N/A, not available; N, no; Y, yes; R, right; L, left.

## Discussion

MET activation is a primary oncogenic driver gene in lung cancer. MET dysregulation, including gene mutation, amplification, and rearrangement, might cause sustained MET activation and oncogenesis. Therapeutics targeting MET have been developed by several agents, including MET TKI, anti-MET, anti-hepatocyte growth factor antibodies, and anti-MET antibody-drug conjugates (6). Among these therapies, preclinical and clinical evidence suggests that MET gene amplification and MET exon 14 alterations are promising prospects for MET inhibitors (12, 26, 27).

Previous studies have shown that the MET exon 14 skipping mutation occurred in approximately 3–5% of lung adenocarcinoma cases, slightly more than 2% of squamous cell carcinomas, and 2.3% of other lung cancer histology types (6, 28, 29). Moreover, the MET exon 14 skipping mutation showed a specifically high incidence in lung sarcomatoid carcinomas (approximately 11–30%) (7, 30). In the present study, the MET exon 14 skipping mutation occurred in 0.90% (41/4,564) of adenocarcinoma, 1.02% (3/294) of squamous cell carcinoma, and 8.33% (1/12) of sarcomatoid carcinoma specimens. The incidence of the MET exon 14 skipping mutation in our medical center was slightly lower than that in other studies, which might be caused by the composition of the tested population at different medical centers. The MET exon 14 skipping mutation was significantly associated with older age (>60 years, *P* = 0.001), and there were no significant differences in sex, tumor stage, tumor location, smoke, pleural invasion, and histology type. These findings are consistent with those of a previous study (31).

MET amplification occurs in 1–6% of patients with NSCLC (12) and is associated with smoking and advanced tumor stage (7, 15). In our data, the incidence of MET amplification was 0.79% (23/2,927) in all types of lung cancers, 0.78% (21/2,676) in adenocarcinoma and 1.07% (2/187) in squamous cell carcinoma, with no MET amplification in other histology types. MET amplification was closely related to an advanced tumor stage (III + IV, *P*=0.000) and showed a trend of higher mutation rate in the right lobe of the lung (*P* = 0.058), which might be due to the anatomical difference of this lobe of the lung that is trilobar and the left lobe is bilobar (32).

Recently, several preclinical and clinical studies have suggested that MET TKIs such as tepotinib, crizotinib, and capmatinib have shown promising therapeutic effects in patients with lung cancer (12, 27, 33). Based on the preliminary data and FDA approval, the National Comprehensive Cancer Network recommended capmatinib, tepotinib, and crizotinib (useful in certain circumstances) as first-line therapy or subsequent therapy alternative for patients with the MET exon 14 skipping mutation. Based on the significant therapeutic effect of crizonib and capmatinib in patients with MET amplification, the National Comprehensive Cancer Network also recommends high-level MET amplification as an emerging biomarker to identify novel target therapies for metastatic NSCLC (12, 34).

The MET exon 14 skipping mutation and MET amplification are independent poor prognosis factors (7). In our research, we attempted to analyze the survival of these patients; however, the number of patients in each group was too small and some patients were recently diagnosed, making it impossible for us to effectively analyze the survival of patients based on this relatively small sample size. We will continue to collect MET genomic alteration cases and analyze the relationship between survival and MET genomic alterations.

Oncogenic gene mutations are usually mutually exclusive in patients with lung cancer. MET genomic alterations do not typically overlap with other genes, such as EGFR, ALK, and BRAF (9, 18, 19). Nonetheless, the MET exon 14 skipping mutation can co-occur with MET amplification (7). In our study, three patients had both the MET exon 14 skipping mutation and EGFR exon 19 19-Del, EGFR exon21 L858R, and BRAF V600E. We also found one case with the MET exon 14 skipping mutation and MET amplification, which was consistent with a previous study (7). One patient with both the MET exon 14 skipping mutation and gene amplification showed a major partial response to the MET inhibitor crizotinib (9). Unfortunately, the patient in our study underwent surgical resection, making it impossible to analyze the response of this patient to MET inhibitors. MET amplification is an important mechanism for first- and second-generation EGFR-TKI resistance, mostly based on the presence of EGFR p. Thr790MET (T790M) (35). In our study, 11 patients were primarily positive for MET amplification and EGFR mutation; however, these patients all underwent surgical resection of the primary tumors and remained without recurrence or metastasis, making it impossible to analyze their responses to EGFR-TKIs in the short term. Our group will continue to track these patients and analyze changes in their future conditions.

In the present study, we also analyzed 35 cases to identify the DNA splicing sites causing mRNA level changes due to the MET exon 14 skipping mutation. We found that SNVs and small fragment deletions led to the mRNA exon 14 skipping mutation. SNV mutations occurred upstream of exon 14, including c.2888-1 G>A (1/35, 2.86%) and downstream of exon 14, such as c.3028G>A (2/35, 5.71%), c.3028G>C (4/35, 11.43%), and c.3028G>T (3/35, 8.57%). Deletion also occurred upstream and downstream of MET exon 14, such as c.2888-13_2908del and c.3027_3028+9del. Our findings provide an interpretation basis for the MET exon 14 skipping mutation using NGS.

In this study, we used DNA-based NGS and RNA-based ARMS-PCR methods to analyze MET exon 14 skipping mutation. A previous study has demonstrated that compared to qRT-PCR and Sanger sequencing, NGS method could be the first choice for multiplex detection due to its high sensitivity and large panel of detection (10). The concordance of MET exon 14 skipping mutation detected by DNA- and RNA-based NGS in pulmonary sarcomatoid carcinomas was 96.1% (36). ARMS-PCR was also considered a reliable gene mutation testing method in lung cancer (37, 38). In our study, we found 27 cases of MET exon 14 skipping mutation by ARMS-PCR and 18 cases by NGS. After cross-validating the genomic alterations in 35 patients, we found that all the patients harbored MET exon 14 skipping mutation; the concordance observed in our center for DNA-based NGS and ARMS-PCR in detecting MET exon 14 skipping mutation was 100%. Although we showed high consistency between these two methods, considering that some genomic alterations uncovering MET exon 14 splice sites might cause skipping mutation, RNA-based sequencing seems to be a more accurate method (10, 36). In our study, most of the specimens were tested within 6 months after paraffin embedding to meet the quality standard. For those less optimal archived tissue samples such as those embedded over 2 years earlier or the proportion of tumor cells was less than 20%, we would first enrich the tumor area and extract the genomic DNA and RNA, check the quality to see if it meets the standard; for samples meeting the standard, we would then continue to conduct the NGS or ARMS-PCR detection.

The role of MET in carcinogenesis and cancer development has been extensively studied. The current challenges are identifying molecular subtypes and subgroups of patients that would benefit most from MET inhibitors or the combination of other genomic target agents. Further extensive studies and clinical trials must be conducted, and clinicians should closely follow up and observe the conditions of specific subsets of patients. We believe that in the next 5 years, as the widespread implementation of high-throughput sequencing and in-depth investigation of the role of MET in cancer occurs, more rare mutation sites will be discovered and more target drugs will be developed to treat patients with more detailed classifications.

There were several limitations to the present study: (1) a single-center study; (2) lack of survival data of the patients; (3) no track of clinical treatment data such as chemotherapy and radiotherapy applications; and (4) no cross-comparison with other mutation genes such as EGFR, KRAS, and HER2.

In summary, we collected and analyzed the genomic alteration of MET in 5,008 lung cancer cases and reported the accurate incidence of MET exon 14 skipping mutation and MET amplification and their correlations with clinicopathological features in patients from east China. We found that the MET exon 14 skipping mutation was significantly associated with older ages, whereas MET amplification showed a higher incidence in advanced tumor stage, providing a valuable reference for clinicians from east China to study this uncommon molecular marker. Moreover, we reported several DNA mutation sites that could lead to the exon 14 skipping mutation at the mRNA level to assist other genetic testing agencies in interpreting their MET genetic testing results.

## Data Availability Statement

According to national legislation/guidelines, specifically the Administrative Regulations of the People’s Republic of China on Human Genetic Resources (http://www.gov.cn/zhengce/content/2019-06/10/content_5398829.htm, http://english.www.gov.cn/policies/latest_releases/2019/06/10/content_281476708945462.htm), no additional raw data is available at this time. Data of this project can be accessed after an approval application to the China National Genebank (CNGB, https://db.cngb.org/cnsa/). Please refer to https://db.cngb.org/, or email: CNGBdb@cngb.org for detailed application guidance. The accession code CNP0002208 should be included in the application.

## Ethics Statement

All patients provided written informed consent, and the Ethics Committee of Qingdao University approved the study (approval NO. QYFY WZLL 26577).

## Author Contributions

YS, GL, and XX conceived and designed the study. XX contributed reagents, protocols, and materials. GL, WR, HZ, GZ, and YC conducted the experiments and analyzed the raw experimental data. YS provided a pathological diagnosis. YS, KJ, MH, YH, and XL collected and statistically analyzed the data. YS wrote the paper. XX modified the manuscript. All authors contributed to the article and approved the submitted version.

## Funding

This work was supported by the Clinical Medicine+X Foundation of the Affiliated Hospital of Qingdao University (YS).

## Conflict of Interest

Author GZ was employed by Geneis Beijing Co., Ltd.

The remaining authors declare that the research was conducted in the absence of any commercial or financial relationships that could be construed as a potential conflict of interest.

## Publisher’s Note

All claims expressed in this article are solely those of the authors and do not necessarily represent those of their affiliated organizations, or those of the publisher, the editors and the reviewers. Any product that may be evaluated in this article, or claim that may be made by its manufacturer, is not guaranteed or endorsed by the publisher.
